# The Reactive Sulfur Species Concept: 15 Years On

**DOI:** 10.3390/antiox6020038

**Published:** 2017-05-23

**Authors:** Gregory I. Giles, Muhammad Jawad Nasim, Wesam Ali, Claus Jacob

**Affiliations:** 1 Department of Pharmacology and Toxicology, University of Otago, PO Box 56, Dunedin 9054, New Zealand; 2 Division of Bioorganic Chemistry, School of Pharmacy, Saarland University, Campus B2 1, Saarbruecken D-66123, Germany; jawad.nasim@uni-saarland.de (M.J.N.); s8wealii@stud.uni-saarland.de (W.A.)

**Keywords:** cellular thiolstat, redox signalling, sulfur radicals, reactive sulfur species, redoxome.

## Abstract

Fifteen years ago, in 2001, the concept of “Reactive Sulfur Species” or RSS was advocated as a working hypothesis. Since then various organic as well as inorganic RSS have attracted considerable interest and stimulated many new and often unexpected avenues in research and product development. During this time, it has become apparent that molecules with sulfur-containing functional groups are not just the passive “victims” of oxidative stress or simple conveyors of signals in cells, but can also be stressors in their own right, with pivotal roles in cellular function and homeostasis. Many “exotic” sulfur-based compounds, often of natural origin, have entered the fray in the context of nutrition, ageing, chemoprevention and therapy. In parallel, the field of inorganic RSS has come to the forefront of research, with short-lived yet metabolically important intermediates, such as various sulfur-nitrogen species and polysulfides (S_x_^2−^), playing important roles. Between 2003 and 2005 several breath-taking discoveries emerged characterising unusual sulfur redox states in biology, and since then the truly unique role of sulfur-dependent redox systems has become apparent. Following these discoveries, over the last decade a “hunt” and, more recently, mining for such modifications has begun—and still continues—often in conjunction with new, innovative and complex labelling and analytical methods to capture the (entire) sulfur “redoxome”. A key distinction for RSS is that, unlike oxygen or nitrogen, sulfur not only forms a plethora of specific reactive species, but sulfur also targets itself, as sulfur containing molecules, i.e., peptides, proteins and enzymes, preferentially react with RSS. Not surprisingly, today this sulfur-centred redox signalling and control inside the living cell is a burning issue, which has moved on from the predominantly thiol/disulfide biochemistry of the past to a complex labyrinth of interacting signalling and control pathways which involve various sulfur oxidation states, sulfur species and reactions. RSS are omnipresent and, in some instances, are even considered as the true bearers of redox control, perhaps being more important than the Reactive Oxygen Species (ROS) or Reactive Nitrogen Species (RNS) which for decades have dominated the redox field. In other(s) words, in 2017, sulfur redox is “on the rise”, and the idea of RSS resonates throughout the Life Sciences. Still, the RSS story isn’t over yet. Many RSS are at the heart of “mistaken identities” which urgently require clarification and may even provide the foundations for further scientific revolutions in the years to come. In light of these developments, it is therefore the perfect time to revisit the original hypotheses, to select highlights in the field and to question and eventually update our concept of “Reactive Sulfur Species”.

## 1. Foreword

Thiology has reached a new level. This tour dʹhorizon on Reactive Sulfur Species, written by the originators of the term and concept, illustrates the substantial increase in knowledge and understanding of the role of thiols and related compounds in redox biology which occurred in the last 15 years. Written in the reader-friendly narrative characteristic of the authors, the field of active research is presented in a perspective pointing towards a rich future in reactive sulfur species (RSS) research: congratulations!—Professor Helmut Sies, Duesseldorf.

## 2. The Importance of Being Yellow

In the field of Redox Biology the year 2016 has marked a very special anniversary. It has been 15 years since we published our paper entitled “Hypothesis: The role of reactive sulfur species in oxidative stress” [1]. In hindsight, this paper heralded what many philosophers in the Kuhnian tradition would probably refer to as a scientific revolution [2] in our understanding of the role of sulfur in biology. Prior to 2001, redox biology was predominantly concerned with metal ions and reactive oxygen and nitrogen species (ROS and RNS). Sulfur was mainly considered as a structural component of proteins and, in the case of glutathione, as a passive redox buffer. In contrast sulfur’s redox partner selenium captured much of the interest of researchers looking beyond conventional redox species, as it was known to be found in a few exotic, yet pivotal, enzymes such as the glutathione peroxidases and the emerging selenoprotein P [3]. Today the picture is entirely different, with reactive sulfur species (RSS) established as key signalling molecules in their own right [4,5,6,7,8]. Yet, once more, change is in the air and the field is again rich in anomalies and controversies. It is therefore the perfect time to revisit and, in light of recent developments, to reconsider the original concept of RSS.

There are various ways to cogitate upon such a topic. One may run a historical review, yet many excellent and up-to-date reviews already exist in this field and we will gladly refer to them in passing [5,6,7,8]. “Special Issues” are already prevalent [9,10] and, while it is also possible to develop a new bold hypothesis [11,12,13], we have decided to take a slightly different approach. We will take this opportunity to celebrate some of the most stunning and innovative developments in RSS during the last 15 years. As we will have limited time on our journey, we will only stop at selected highlights of mammalian biochemistry, and reserve some consideration for the emerging fields of dietary, nutraceutical and pharmaceutical RSS. We will also cover the emergence of various concepts that have accompanied these developments, such as the sulfur redoxome, the sulfenome, ultra-exogenous sulfide formation, and the cellular thiolstat [12,13,14,15,16]. Based on those highlights, and the numerous questions they have raised, we will eventually attempt to define a modern, operable definition of RSS, which we will develop step by step towards the end of this manuscript.

## 3. Do You Remember the Time?

### 3.1. A Historical Perspective

At the beginning of the Millennium, the field of oxidative stress research was dominated by ROS and RNS. Alongside the highly visible and increasingly prominent hydrogen peroxide (H_2_O_2_), diverse oxygen-centred radicals, such as the superoxide (O_2_^•−^) and hydroxyl (^•^OH) radicals, were recognized as important oxidative stressors, while a range of nitrogen-oxygen species such as nitric oxide (^•^NO) and peroxynitrite (ONOO^−^) could also cause their own specific cellular damage [17]. With a few notable exceptions, such as lipid peroxides (ROOH), most of these species were small inorganic molecules with short half lives. Hence when the authors originally conceived that there may be analogous sulfur-centred reactive species in biology, they instinctually turned to similar small sulfur molecules first—only to become terribly disillusioned.

**Figure 1:**
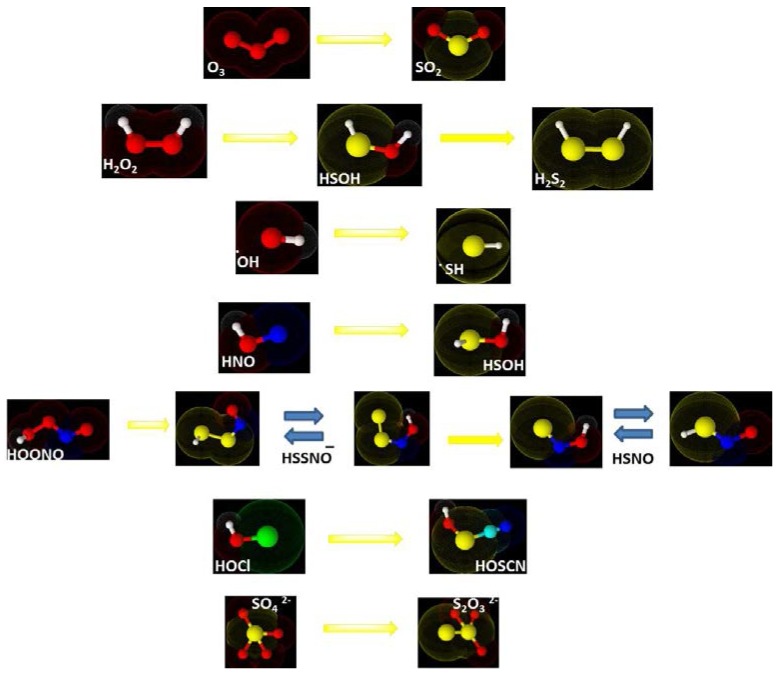
A selection of RSS derived by simple analogy with ROS and RNS. This kind of “isosteric replacement” of sulfur atoms for oxygen, and on occasion for nitrogen, is a theoretical exercise on paper. It leads to a range of rather exotic RSS which only recently have been discovered in the context of human biology and often are still controversial.

Certain sulfur compounds, such as sulfur dioxide (SO_2_), were known as environmental toxinst—and at the time the same applied to hydrogen sulfide (H_2_S). SO_2_, for instance, could be seen as a sulfur analogue of ozone, O_3_, yet such a simple isosteric replacement of oxygen (or nitrogen) for sulfur would not take us far, at least not 15 years ago (Figure 1). H_2_S as analogue of H_2_O is nonsense as water is not a ROS, H_2_S_2_ as analogue of H_2_O_2_ would have been perfect as we will see—yet was not accepted among the biological sulfur redox community at the time, and HSNO as relative of HOONO would also have worked if that molecule only would have been known at the time (see Section 5). Other sulfur species, such as certain inorganic thiocyanates and oxidized forms of thiocyanites, were known already and appeared to possess a certain biological relevance, yet they were more or less confined niche publications. Still other sulfur compounds were considered, such as Caro’s acid (peroxosulfuric acid, H_2_SO_5_), but these eventually turned out to be probably irrelevant in biology. Eventually, the attempt to construct a concept of RSS based on an analogy between ROS and RNS was rather futile. Inspiration therefore had to come from other quarters.

### 3.2. Inspiration from Natural Products

With around 140 g in a healthy human adult with average weight of 70 kg, sulfur is hardly a trace element [18] and was known to exist in various organic molecules, including the amino acids cysteine, methionine, homocysteine and taurine. In addition to these species with well defined sulfur oxidation states, sulfur can also occur in different and often unusual oxidation states. At the turn of the Millennium H_2_S, thiols (RSH), disulfides (RSSR’) and sulfate (SO_4_^2−^) had already been known in many different biological contexts for years. Throughout the 20th Century redox active RSS, such as sulfur-centred radicals, reactive disulfides, sulfenic acids and some more exotic sulfur species had been identified and characterized as the result of studies on thiol oxidation in glutathione and proteins, as well as attempts to understand the actions of naturally occurring Organic Sulfur Compounds (OSCs) [19,20]. Research into the thiols as quenchers of singlet molecular oxygen in 1991, for instance, shed light on the existence of RSS a decade before the RSS concept became prominent [19]. Similarly, the catalytic cycles of the human thioredoxin reductase or the various bacterial NADH (per)oxidases contain some intriguing sulfur modifications, which in hindsight may well be described as—resembling—RSS. It is perhaps symptomatic of the maturity of the field in the early 2000s that certain reviews were published on this facet-rich topic at around the same time [21].

The prominent role of glutathione as cellular redox buffer during oxidative stress had also been known for a while, yet its role as a key regulatory post-translational modification in proteins, and hence as signalling molecule involved in *S*-thio(ny)lation and dethio(ny)lation processes comparable to phosphorylation/dephosphorylation or acetlyation/deacetylation, was just emerging [22,23,24]. Similarly sulfur-based oxidative stressors were known and well characterized, for instance sulfur-based radicals [25,26] which are experiencing a renaissance today as part of the ongoing controversy on one versus two electron reactions and kinetic issues [27]. Other molecules suspected of being RSS had to be borrowed from the plant kingdom [28]. There, chemically exotic species such as the disulfide-*S*-oxides RS(O)SR’ and RS(O)_2_SR’, which we now refer to as thiosulfinates and thiosulfonates, had been known for decades [29] and their extraordinary chemistry was a true inspiration for the potential role of RSS in biology.

2001 was therefore not the beginning of RSS research. Let us consider Table 1, which is adapted from our publication in 2001 and lists some of the most prominent RSS discussed at that time [1]. It is immediately apparent that this list contains predominantly organic molecules, and that the analogy with either ROS or RNS is still weak. Whilst this list was fairly up-to-date in 2001, even daring in parts, today it appears like a small fragment from the past. Still, it already contains some of the seeds for future diversification in different branches of redox chemistry, biochemistry, biology, nutrition and pharmacy, and hence also for controversies as to the exact nature of RSS. At the beginning of the 21st Century many of these species had not been conclusively identified in mammalian biology, and in any case they were not deemed as particularly important. During the years that followed this abruptly changed, opening up the extensive possibilities inherent in sulfur chemistry, with investigators now primed to expect the unexpected [12].

## 4. The Year 2003: Gestalt Switch in the Field of RSS Research

**Figure 2:**
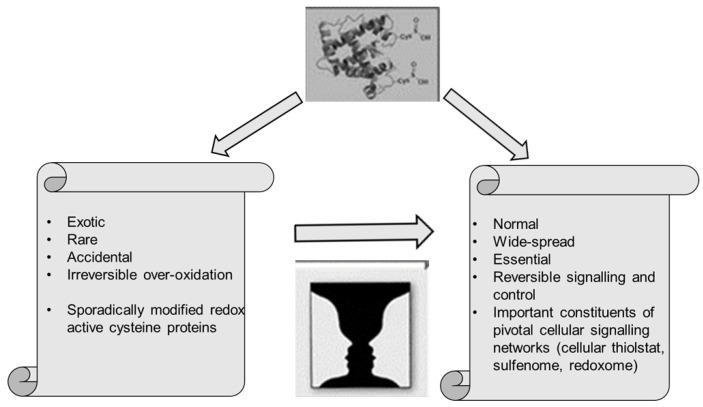
Schematic illustration of the Gestalt switch which occurred in the field of sulfur redox biology during the first decade of this century. This conceptual switch has been a prerequisite to bring sulfur redox systems to the forefront of widely accepted redox research, and to stimulate new projects, concepts, and even the emergence of new or upsurge of existing journals in this field.

Undoubtedly, the most remarkable discoveries in the years that followed 2001 concerned cysteine proteins and enzymes. We will therefore initially focus on those particular developments, bearing in mind that parallel strands of investigation, for instance in the field of H_2_S signalling or nutrition, were of equal importance. The discoveries of new cysteine modifications in proteins, however, bore certain hallmarks of a major breakthrough. As we will see, the “logic” behind that breakthrough follows the traditional epistemological view that mounting anomalies in a field of science may sooner or later result in a scientific revolution, in a change of paradigms culminating in a “Gestalt switch” when it comes to interpret previous data, and eventually the emergence of new scientific communities with their own mode(s) of communication (Figure 2).

### 4.1. Sulfinic Acids in Proteins and Enzymes: Accidental or with (a) Purpose

In 2000, Jennifer Littlechild and her colleagues reported the “crystal structure of decameric 2-Cys peroxiredoxin from human erythrocytes at 1.7 Å resolution” [30]. In this paper, the authors noted that the catalytic cysteine residue responsible for peroxide reduction was oxidized to a cysteine sulfinic acid. Today, in the age of floodgates, sulfenomes and redoxomes, such a statement sounds fairly trivial. Yet in 2001, the idea that a sulfinic acid may occur in an enzyme such as peroxiredoxin (Prdx), still caused considerable controversy, and suspicion that this overoxidation was an artefact due to a prolonged crystallization procedure. In 2001, and from a more teleological perspective, one would also have questioned the evolutionary benefit behind the formation of a sulfinic acid in Prdx, as the protein bearing this modification would be irreversibly inactivated as a redox enzyme [31].

A discovery soon thereafter in 2003 changed this perspective, and in many ways turned the idea of sulfinic acid formation in proteins upside down, resulting in the Gestalt switch. Three years after the report of the crystal structure of Prdx, and two years after our hypothesis of RSS, Michael Toledano and his colleagues reported in Nature the discovery of the protein sulfiredoxin (Srx) in yeast, which apparently was able to reduce the sulfinic acid found in overoxidized Prdx back to the normal sulfenic acid and thiol oxidation states [32]. Suddenly, the overoxidized sulfinic acid was no longer an accident, or even artefact, but a clever way to switch the enzyme on and off as part of a complicated redox sensing and feedback circuit.

This discovery had significant implications, not only for yeast Prdx, but also for our entire understanding of sulfur redox biology. First of all, it confirmed that unusual sulfur modifications, such as sulfinic acids, may indeed occur widely in (eukaryotic) biology, not as accidents, irreparable damages caused by ROS/RNS or, one forbids, experimental artefacts, but as normal modifications with essential sensing, signalling, feedback and control functions. These modifications may be rather abundant in proteins and enzymes, a suspicion which soon thereafter would give rise to extensive hunting and mining for such modifications within the proteome. Secondly, the discovery of Srx and its interactions with Prdx broke down previous concerns about the existence of unusual post-translational sulfur modifications in proteins, and therefore also paved the way for research into other unusual sulfur species, such as persulfides or trisulfanes, which appeared on the scene a few years later on. Indeed, the interaction between Srx and Prdx is now known to involve a considerable number of truly exceptional sulfur modifications, from the better known thiol, disulfide and sulfenic acid oxidation states, to the sulfinic acid and, new on stage, a highly unusual phosphorylated sulfinic acid intermediate (sulfinic acid phosphoryl ester, Prdx–Cys–S(=O)OPO_3_^2−^) and, finally, a disulfide-*S*-monoxide (thiosulfinate, Prdx–Cys–S(=O)–S–R) [33].

Here one should note the difference: the same data as in 2000, but an entirely different view on it. In hindsight, many of the rather surprising, and perhaps questionable, findings reported in the years prior to 2003 suddenly became obvious, acceptable and evident. Indeed, as an indication of the incredible rate of progress in this field once the initial dam was breached, a publication a year later describes the cysteine sulfinic acid driven mitochondrial localization of the Parkinson disease protein DJ-1, dealing with this underlying cysteine modification as if it were mainstream, without considering a sulfinic acid modification as a particularly unusual step in protein signalling [34].

### 4.2. New Paradigms: Floodgates, Signals and Omens of Omes to Come

Driven by the discovery of Srx and its impact on Prdx, and due to its biochemical, but especially also conceptual importance, 2003 and 2004 witnessed the developments of the first new concepts in the field of sulfur-based cellular redox control, from sensing and feedback to control and signalling.

In 2004, some of us discussed the sulfinic acid switch in proteins in Organic and Biomolecular Chemistry, an idea which subsequently gathered steam and, in 2011, led to the coining of the expression cellular thiolstat [15,35]. This concept assumes that the cysteine proteins and enzymes in cells are sensors of oxidative stress, and that their sequential and often reversible oxidative modification during an oxidative onslaught triggers widespread cellular signalling which enables the cell to respond adequately, either by mounting an antioxidant defence or by shutting down via apoptosis [5]. In subsequent years, numerous studies have confirmed this idea of a gradual, measured and often complicated and cell type-specific response conveyed by cysteine proteins with different reactivity or sensitivity towards different oxidative modifiers [28,36,37,38,39,40].

Other colleagues, such as Zachary Wood, Leslie Poole, Andrew Karplus and Al Claiborne, were soon considering the wider implications of the sulfenic and sulfinic acid protein chemistry on redox signalling with their floodgate model [41,42,43]. The latter was developed in the context of Prdx and published in 2003 as “Peroxiredoxin evolution and the regulation of hydrogen peroxide signaling” in Science [41]. Initially the authors still considered the oxidation of sulfenic to sulfinic acid as irreversible, yet this one-off switch-off soon turned into a more flexible tool in the context of “Protein sulfenic acids in redox signalling”, published in Annual Reviews in Pharmacology and Toxicology in 2004 [42]. These considerations would later contribute to the idea of a wider cellular redoxome proposed by colleagues such as Michel Toledano and Ursula Jakob in 2010 and 2011, respectively [12,13]. As we will see, mining such a redoxome with proteomic tools has subsequently become an interesting activity, and the nuggets of information found seem to lead to a system of cell signalling revolving around sulfur based redox systems [11].

In the years that followed, the field of unusual sulfur modifications in proteins and enzymes gathered considerable steam, fuelled in part by new analytical, often proteomic methods employed by colleagues seeking to unravel the secrets of RSS in cell biology. This field has witnessed considerable progress, especially during the last decade. Today, we are slowly becoming aware of the full extent of redox signalling based on thionylation and glutathionylation, processes which have been described by authors such as Pietro Ghezzi over ten years ago in landmark publications such as “Regulation of protein function by glutathionylation” or “Oxidoreduction of protein thiols in redox regulation”. Indeed redox modifications are now seen to be of comparable importance to phosphorylation and dephosphorylation [22,24]. Coincidentally, this field of signalling research has also seen significant changes in and around the year 2003, for instance with the publication of “The changing faces of glutathione, a cellular protagonist” by Alfonso Pompella and colleagues appearing in that year [23]. At this time, the notion of GSH as antioxidant slowly began to give way to a more sophisticated view of the regulatory role of this tripeptide, for instance as a means of protein post-translational modification via cysteine *S*-glutathionylation, and as a source of RSS.

Within this tradition “Redox regulation of cell contacts by tricellulin and occludin (…)” is just the latest discovery in a field of sulfur-based redox regulation and signalling dominated by the—usually reversible—formation of disulfides [44,45]. Yet more exotic and apparently less widespread modifications, such as sulfenic acids, trisulfanes (RSSSR’) and even sulfane sulfur (RSSH) also have been found and seem to play some vital role in sulfur redox biology (see below) [1,21]. Eventually, the field of sulfur redox biology has moved on considerably from the few examples initially found to be redox regulated in this fashion, such as Prdx, and today includes a wide palette of proteins and enzymes and—often controversial—concepts, such as the sulfenome and sulfur redoxome put forward in 2009 and 2010, or the cellular thiolstat in 2011 [11,12,15].

After this brief detour to the field of post-translational cysteine modifications, which has shed much light on the possible redox states that may be found in RSS, we will now return to the key issues surrounding RSS and refer to the many excellent reviews that already exist on this topic for further information [1,5,46]. We should note that those cysteine modifications in proteins probably do not count as RSS in a narrow sense, but as conveyors of signals. Those signals may well be triggered by RSS, and later on we will need to come back to the issue of sulfur being at the same time part of reactive species and of their targets. Still, we should realize that the various cysteine modifications have to some extent inspired research into RSS. They have served as footprints for the presence of RSS, e.g., persulfides in proteins as indicators for the action of polysulfides, or have assisted as blueprints in the design of specific thiol modifying agents, e.g., sulfenic acids or disulfide-*S*-oxides as selective modifiers of target proteins. Ultimately, the developments in the field of sulfur protein redox biology have also raised interest and stimulated research and in the field sulfur redox biology in general.

## 5. What are RSS?

**Figure 3:**
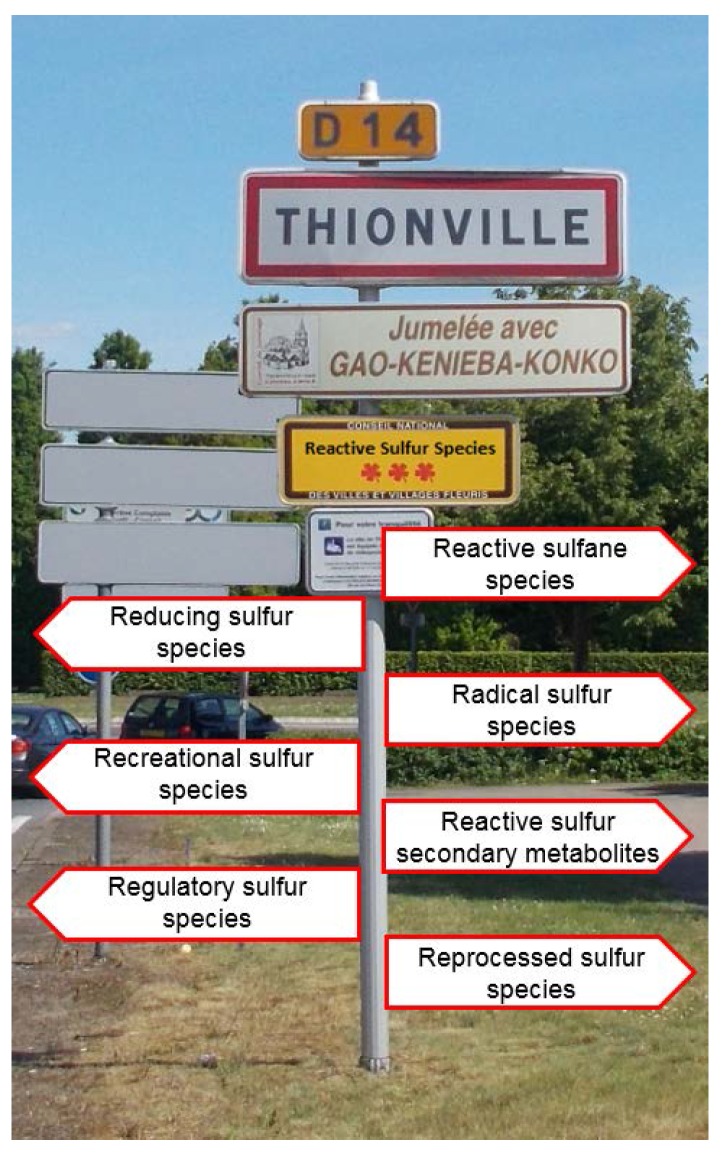
The diversification of the concept of RSS into different directions, from “Regulatory Sulfur Species” and inorganic “Reactive Sulfide Species” to “Recreational Sulfur Substances” used in various remedies. This diversification often depends on the scientific communities involved and is not necessarily mutually exclusive. The signpost contains real, published and simply by us invented malapropisms of the original RSS abbreviation, and is superimposed on a real signpost of the town of Thionville in Lorraine (France).

As we have seen before, the original concept of RSS was surprisingly vague on the exact nature and definition of RSS. This inherent uncertainty at the time was due to the fact that only a few true analogues of ROS or RNS had been identified in biological systems, that most molecules known in the field were either found in secondary metabolites from plants, fungi or lower organisms, or as rare post-translational cysteine modifications in proteins and enzymes, and that sulfur was traditionally thought to act purely as an antioxidant. Therefore an open and flexible definition of RSS was desirable for the new concept to expand and prosper. Such an intended vagueness early on has, however, also provided the seeds for future controversies as to the definition of RSS. Are RSS organic or inorganic sulfur species? Are they radicals or rather non-radicals, for instance electrophiles? Are all RSS oxidants or are (some) RSS reductants? Are RSS formed endogenously inside the body as part of redox biology or are they exogenous species, for instance from the food or air? Are RSS first-line redox modulators or simply victims or follow-on products of the action of ROS or RNS? And, eventually, are all RSS redox active, or are some RSS also active by rather binding to metal ions or metalloproteins? We will take advantage of these contrasts and consider some of the current RSS concepts, where the letters R, S and S by intent may no longer stand for “reactive”, “sulfur” and “species”, as shown in Figure 3, in the hope to illuminate some of the underlying issues and to eventually arrive at a more harmonious working definition.

### 5.1. RSS: Reactive Sulfane Species as Organic Signalling Molecules and Secondary Metabolites

Apart from some notable exceptions, such as lipid peroxides (ROOH), the majority of ROS and RNS are inorganic species. In the case of RSS, the matter is more complicated. Whilst there are some better known inorganic sulfur species that one may well consider as RSS, such as sulfur dioxide (SO_2_), some inorganic polysulfides S_x_^2−^, or—more controversially—thiosulfinate S_2_O_3_^2−^, these molecules traditionally have not been at the forefront of redox biology. Instead, we find a whole arsenal of organic sulfur species, from small molecules formed in mammals such as GSSG, GS(O)SG, GSSG^•−^ and GSSH, to an almost inexhaustible variety of sulfur based secondary metabolites—one only has to remember garlic—and the same also applies to fungi and bacteria.

It is therefore virtually impossible to provide a comprehensive list of organic sulfanes. Still, based on the notion that those molecules usually contain at least one carbon-sulfur bond, we can select some of the most prominent species, which include sulfenic and sulfinic acids, but also organic disulfanes (RSSR’, for instance GSSG), polysulfanes (RS_x_R’, x ≥ 3), thiosulfinates, thiosulfonates, 1,2-dithiole-3-thiones and similar molecules. A recent perspective article by Ruma Banerjee in Nature Chemical Biology on the “Biogenesis of reactive sulfur species for signalling by hydrogen sulfide pathways”, although focussed on the emerging story and stories surrounding H_2_S and its oxidized follow-on products, provides an excellent and literally colourful overview of some of the most prominent RSS [47]. We would also like to draw particular attention to the diverse and often controversial use of the “correct” sulfur nomenclature, in organic as well as inorganic species, and a fairly comprehensive summary on this issue in a recent publication of John Toohey and Arthur Cooper [48]. Within this context, we need to mention that the nomenclature for inorganic and organic polysulfides and polysulfanes is still not settled satisfactorily, and that we preferably use the sulfane terminology for organic, uncharged species and the sulfide terminology for molecules such as RS_x_H or inorganic H_2_S_x_, i.e., sulfur compounds which may form charged, anionic species.

Logically and historically, a consideration of inorganic and organic RSS starts with H_2_S and the modifications it may give rise to in organic or inorganic molecules. Paralleling the discoveries of highly oxidized forms of sulfur being found in proteins as described above, at around the same time sulfur in its reduced form began the transition from a noxious gas with the stench of rotten eggs to a key signalling molecule. Following on from an early report by Lorne R. Goodwin that sulfide could be found in post mortem human brains, suspicions slowly began to emerge in the literature that sulfide, or sulfide derivatives, could be endogenous neurotransmitters [49]. At the time this was considered infeasible, as gaseous H_2_S was known to be a highly potent toxin, causing adverse effects at 50 ppm and fatality at 700 ppm [50]. With the discovery that the enzymes cystathionine β-synthase and cystathionine γ-lyase, which generate H_2_S from cysteine, could be found in various tissues, however, the hunt was on to detect H_2_S in mammalian biochemistry [51]. Soon Dave Kraus and colleagues were developing methodologies to enable them to sense this newly discovered signalling molecule [52], with the late 1990s and early 2000s witnessing the first reports that endogenously generated H_2_S acts as a neurotransmitter [53,54], vasorelaxant [55,56] and inflammatory mediator [57].

In a similar manner to the challenges raised by the range of oxidation states accessible to protein thiols, the reactivity of H_2_S has both enriched and complicated this field. Different assays disagree on the actual level of “free” H_2_S present in tissues, as downstream RSS formed from H_2_S oxidation, as well as RSS-protein adducts, are frequently detected and misreported as H_2_S, while H_2_S itself is volatile and its solution concentration can change within minutes of tissue extraction [58]. Undoubtedly there remains much to be determined in establishing the respective role of H_2_S versus subsequent organic RSS formation in cell signalling.

Intriguingly, organic RSS are not only formed by the reaction with H_2_S, but also by the oxidation of thiols (RSH). Such organic RSS are found, for instance, as part of complex metabolic pathways as well as being generated by more or less unspecific interactions with ROS and RNS. The formation of unusual sulfur species during the reaction of GSH with H_2_O_2_ has been reported several decades ago [59]. Similar to ROS and RNS, some of these oxidized sulfur species serve as part of the host defence in plants and lower organisms, such as the thiosulfinate allicin, which is produced enzymatically from a fairly harmless cysteine sulfoxide precursor after a carbon-sulfur bond has beencleaved (C-S-lyase activity) and the resulting sulfenic acids have condensed by chemical reaction in a non-enzymatic step. Yet organic RSS differ considerably from ROS and RNS, not only because they are considerably more diverse with regard to their chemistry and oxidation state. We have just mentioned host defence. Whilst ROS are formed and used in virtually all cells, from plants and microbes to humans, the formation of defensive RSS seems to be more limited to plants and microbes. We will discuss this apparent asymmetry in the occurrence of RSS later on, also with sight on the issue of aerobic versus anaerobic lifestyles and the possibility of RSS formation by the gut bacteria. Furthermore, RSS are also considerably more selective for their targets. Indeed, most RSS act as electrophiles and many react selectively with thiol groups, either in GSH or in proteins, and occasionally also with selenols. Hence the formation, purpose, chemistry, reactivity and ultimately biological activity of (organic) RSS vary significantly from both ROS and RNS. This does not, however, prevent interactions between these species, which eventually may give rise to additional RSS, for instance HSNO or various radicals, sulfur-based acids and disulfide-*S*-oxides.

### 5.2. RSS: Regulatory Sulfur Species Occurring in Proteins and Enzymes

One may, of course, disagree that RSS solely comprise small-molecule organic RSS, and point towards the wide variety of different, and often diverse, post-translational sulfur modifications which during the last ten to fifteen years have been identified in numerous proteins and enzymes as a result of ROS and RNS action. For instance, what about the “Widespread sulfenic acid formation in tissues in response to hydrogen peroxide”, which has been postulated by Philip Eaton and his colleagues in late 2004 [60]? Or the “Sulfenome mining in Arabidopsis thaliana” by Marc de Montagu, Joris Messens and Frank van Breusegem ten years later, just before Vinayak Gupta and Kate Carroll deliberated once more on “Sulfenic acid chemistry, detection and cellular lifetime” [61,62]. In addition, sulfenic acids in proteins are not the only reactive, sulfur-centred post-translational modifications. For example, Luis Romero and his colleagues have identified “*S*-Sulfhydration: A cysteine posttranslational modification in plant systems” [63] in 2015 and Peter Nagy and his colleagues have seen that “A novel persulfide detection method reveals protein persulfide- and polysulfide-reducing functions of thioredoxin and glutathione systems” in 2016 [64].

There are others, of course, such as the more traditional *S*-thio(ny)lation or *S*-glutathio(ny)lation, which play a pivotal role in cell signalling, the equally unusual and intriguing fact that “Redox regulation of protein tyrosine phosphatase 1B involves a sulphenyl-amide intermediate” [20] reported in the year of destiny 2003 or, more recently, “The physiological role of reversible methionine oxidation” [65]. Indeed, “Methionine oxidation and reduction in proteins” [66] has just been discussed by Rodney Levine and colleagues in considerable detail, and it is to some extent reversible. Therefore sulfoxides and sulfones may well also serve as reactive sulfur signalling molecules in their own right. Not surprisingly therefore, the Belgian proteome miners have returned to their shaft and are already shedding light on “Protein methionine sulfoxide dynamics in Arabidopsis thaliana under oxidative stress” [67].

These studies demonstrate the stimulating and amazing results that are currently being generated at a breath-taking pace. During the last couple of years, numerous new analytical techniques have emerged to assist in the detection of such sulfur-centred (cysteine and methionine) post-translational modifications. In contrast to the routine methods used to detect non-reactive protein modifications, however, RSS quantification is often complicated. For instance, methodologies have been devised based on selective fluorescent markers (such as reactive disulfides for thiols, dimedone derivatives for sulfenic acids, etc.), isotope labelling, complicated sequences of sample quenching, reduction/oxidation and subsequent derivatization, or even on intracellular sensors such as green fluorescent proteins (GFPs) or yellow fluorescent proteins (YFPs). Such techniques inevitably have their own inherent limitations and have recently led to considerable controversies, for instance in the case of the mitoflash probe [68,69,70,71]. In order to gain a more global picture, proteomic mining (see above) is currently an alternative, equally state-of-the-art technology in this field. A recent review by Ming Xian, Christopher Chang and colleagues on a “Chemical probe for molecular imaging and detection of hydrogen sulfide and reactive sulfur species in biological systems” provides an excellent overview of the different tools nowadays available in such an analytical mining toolbox [7].

Yet this title also raises a number of important questions with regard to an operable definition of the kind of RSS mentioned therein. Or in other words, are the post-translational modifications of cysteine and methionine residues in proteins, which over the years have been identified by various eloquent mining activities, indeed RSS in a practicable sense, or are they rather aspects of wider antioxidant defence and signalling cascades? For comparison: we do not refer to phosphorylation or acetylation as the products of “Reactive Phosphorous Species” or “Reactive Acetylating Species”. Hence where should we draw the boundary between reactive species and their action and more controlled signalling? Here, sulfur biochemistry is exceptionally complicated, as some of the modifications just discussed may indeed be formed by chemical attack of reactive species, such as sulfenic and sulfinic acids or methionine sulfoxides in the presence of H_2_O_2_, yet they are subsequently “resolved”, i.e., reduced to their original form, by enzymatic action. Hence naïve comparisons with ROS or classic signalling via phosphorylation clearly fall short. This issue is contentious and we will return to it in the context of demarcation for a more contemporary and operable definition of RSS. At this stage, we will emphasize once more the importance of organic RSS, remember that these RSS differ from ROS and RNS—and hence may require a more complicated definition—and refer to the many excellent, recent and very comprehensive reviews that exist already on this topic [1,5,46,72].

### 5.3. RSS: Reactive Sulfide Species from Inorganic Chemistry

If one really focuses on the analogy of between ROS and RNS on one side and RSS on the other, one ends up with a rather curious situation. When we were first considering the idea that, apart from oxygen and nitrogen, sulfur and selenium, as well as perhaps phosphorous, could and should give rise to classes of reactive species, our focus was firmly on inorganic species, yet the search for such species in the late 1990s ended up with a limited number of sulfur radicals, and a handful of oxidizing sulfur compounds, which were ‘cool’ from a chemical perspective (such as Caro’s acid), and ‘hot’ as far as their reactivity was concerned (such as SO_2_ and its environmental impact as acidic rain), yet clearly not that common or useful in a more biological context. “Going organic” was therefore a reasonable strategy at the time, yet it also broke with the original idea of RSS as the sulfur equivalents of ROS and RNS.

It is therefore rather amazing—and in many ways very fortunate—that the field of inorganic RSS over the years has developed its own dynamic, fuelled by three parallel developments: (a) the stream of ten prominent publications on inorganic RSS by Michael Ashby and his colleagues between 2004 and 2010, all with a title commencing with “Reactive Sulfur Species” and focussed on inorganic RSS [46,72,73,74,75,76,77,78,79,80,81], (b) The eruption of H_2_S research, accompanied by the suspicion that H_2_S is probably not really the signalling molecule one had previously thought, but that there is a case of mistaken identity, involving an entire series of inorganic polysulfides (H_2_S_x_), which clearly belong to the realm of inorganic RSS, (c) the issue of the lost electron, i.e., the rather curious situation that many interactions involving thiols and radicals, for instance in the field of S-nitrosothiols, do not add up when it comes to electrons, hence questioning if RSS are really predominantly electrophilic compounds, such as sulfenic acids, or rather radical species. We will now consider the inorganic side of sulfide species in more detail.

The research by Michael Ashby and his colleagues at the time was concerned with rather exotic inorganic molecules such as hypothiocyanite (HOSCN), which is chemically similar to hypochlorous acid (HOCl) and, similar to HOCl and many ROS, is used as part of the body’s inorganic defence force [46,73,74,78]. Taking a more physico-chemical approach, and often focussing on kinetics, these studies were able to identify a range of reactive species, biologically relevant reactions and reaction products related to the concept of (inorganic) RSS. Those studies are particularly noteworthy as they employ innovative spectroscopic techniques to catch transient reactive intermediates, map out reaction cascades and often focus on kinetic aspects, on the question how fast reactions proceed and, in essence, who blinks first (and how much) [46,73,74,76]. Today, those questions are at the centre of (sulfur) redox biology, as many possible reactions considered from a purely thermodynamic perspective are probably not relevant due to slow kinetics. When compared to older publications, the ever-present tables with kinetic parameters in the modern literature on this topic underline the importance of such kinetic considerations, without, of course, negating the role of thermodynamic parameters such as reduction and oxidation potentials.

At around the same time, but in entirely different quarters, evidence began to mount for the existence of reactive, small molecule sulfur-nitrogen species. In 2006, a very interesting paper by Matt Whiteman and colleagues appeared which provided “Evidence for the formation of a novel nitrosothiol from the gaseous mediators nitric oxide and hydrogen sulphide” [82]. At the time, there was convincing evidence for the formation of such an inorganic sulfur-nitrogen species, yet this evidence was indirect and could not support unequivocally a particular chemical structure. At first sight, this may be surprising as thionitrous acid (HSNO) is easily scribbled down on a piece of paper as possible suspect. Indeed, just one year prior to this more biological publication, the group of Alejandro Toro-Labbé had published “On the mechanism of hydrogen transfer HSCH(O)/(S)CHOH and HSNO/SNOH reactions”, a more theoretical study which nonetheless discussed in some detail the “chemistry” of HSNO [83]. Yet as we will see later on, the formation of this simple molecule from H_2_S and ^•^NO does not add up electronically and matters are more complex. The “chemical characterization of the smallest S-nitrosothiol, HSNO (…)” was eventually reported in 2012 by a team around Milos Filipovic [84]. The same group has also proposed a heme-iron-catalyzed mechanism for the generation of HSNO and nitroxyl (HNO), which eventually accounts for the correct electron count [85]. In time, this inorganic RSS chemistry may further support the idea that sulfur acts via one-electron reactions and radicals, such as HS^•^ radicals (see below).

Intriguingly, and within the same context, a similar “reactive sulfur nitrogen species” was postulated in 2014 by an international group led by Martin Feelisch [86]. This nitrosopersulfide anion (SSNO^−^) seemed to be a more stable intermediate following the thionitrite anion (SNO^−^) in a cascade of unusual RS(N)S explaining eventually the sustained bioactivity of ^•^NO. We have seen this species before, in Figure 1, and as part of a rather futile early attempt of isosterically replacing sulfur for oxygen in peroxynitrite (ONOO^−^). If we recall the theoretical publication by the group of Alejandro Toro-Labbé on this issue, i.e., that HSNO and SNOH are tautomers, we may also understand that today, there is some controversy on the true nature of the biologically relevant smallest S-nitrosothiol. Some recent publications in this field, often ending with question marks in the title, and different chemical names for the same species (e.g., nitrosopersulfide and perthionitrite for SSNO^−^) nicely illustrate that those issues are not entirely resolved yet, and that the battle for the genuine RSS resonates on [87,88].

Eventually, many of these species are fairly short-lived, difficult to identify with certainty in intact biological systems and often only accessible via spectro(photo)metric methods. Still, those species attract some interest, for instance for computational investigations “on the possible biological relevance of HSNO isomers” published by Qadir Timerghazin and his colleagues in 2014 [89]. There have also been a number of follow-on physico-chemical publications in the field in an attempt to track down the species involved [90,91]

**Figure 4:**
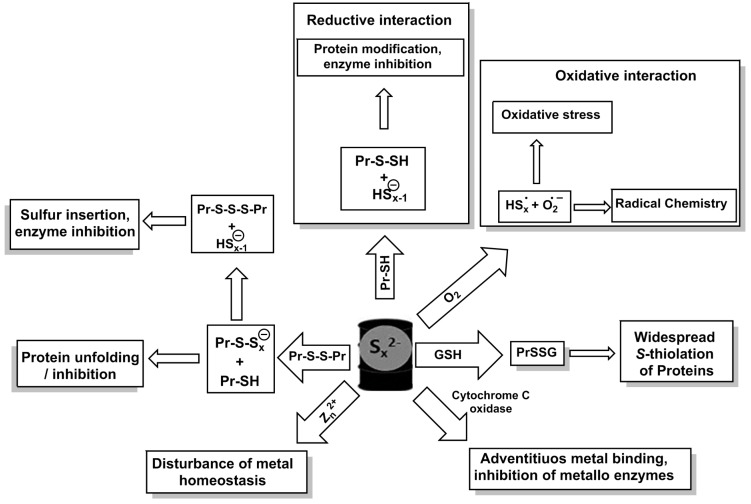
Despite the unassuming chemical composition of inorganic polysulfide S_x_^2−^ species, the reactivity underlying their various biological activities is facet-rich and highly complex. This figure has been adapted from one of our publications on this inorganic matter in 2007 and has been updated to account for the most recent developments in this field, such as HSNO formation [95].

Indeed, fundamental physico-chemical considerations, accompanied by a crucial evaluation of the commonly, routinely employed staining methods for ROS have also led colleagues such as Kenneth Olson to pose the rather provoking question: “A case of mistaken identity: Are Reactive Oxygen Species actually Reactive Sulfide Species?” [72]. In this landmark publication, the authors convincingly demonstrate that many chemical stains considered selective for certain ROS are actually considerably more reactive with—and hence specific for—inorganic RSS, such as the above mentioned polysulfides S_x_^2−^ (Figure 4). Bearing in mind that colleagues such as Hideo Kimura, Jon Fukuto, Tobias Dick and Takaaki Akaike have recently demonstrated the presence of such polysulfides in animal cells, either as products of H_2_S oxidation or, more extreme, as the genuine products of enzymes thought to produce H_2_S, and that a former co-worker of Michael Ashby, Peter Nagy, together with Elias Arnér and colleagues, has just published “a novel persulfide detection method” to explore in more detail the molecular basis of issues surrounding H_2_S signalling, the plot of mistaken identities thickens [64,92,93,94]. As in many instances in RSS research, the words of Louis Pasteur (1822–1895) that “in the field of observation, chance favours only the prepared mind” also holds true here.

This field of RSS research is moving at enormous speed and it is impossible to catch all the recent developments and discussions. We therefore kindly refer to two reviews on this theme, one by Hideo Kimura, the other by Jerzy Bełtowski, both published in 2015 [96,97]. Even so, we envisage that in line with to the above-mentioned Gestalt switch, which took place in sulfur protein redox chemistry around 2003, we are probably about to witness a similar revolution in the field of inorganic sulfur biochemistry. It seems that inorganic polysulfides (S_x_^2−^) may well emerge as the main players in the field of H_2_S-signalling, possibly even eclipsing the role of H_2_S itself. At the same time, they may be responsible for many actions previously ascribed to ROS, from cellular stresses and signalling all the way to the fluorescent signals one may have mistakenly considered as indicative of (certain) ROS. The discovery of “protein persulfide- and polysulfide-reducing functions of thioredoxin and glutathione systems” points into that direction [64].

### 5.4. RSS: Reducing Sulfur Species with Biological Activity

Eventually, the emergence of the polysulfides as standard bearers in H_2_S research may also have a beneficial side effect for our definition of RSS, as this possibly removes the most prominent representative of a class of reducing RSS, i.e., H_2_S itself, from the scene. As mentioned already, polysulfides are reducing as well as oxidizing, yet in biology, they seem to act primarily as oxidants. It is therefore safe to consider RSS generally as reactive, oxidizing sulfur species, yet explicitly without denying the importance of reducing sulfur species and any reductive stress they may be involved in. Here, H_2_S, but also certain other reducing sulfur species may be involved. This matter is particularly complex once those species enter (catalytic) redox cycles, act via non-redox reactions, such as metal (protein) binding, or cause damage by reducing essential disulfide bridges. Traditional thiol-based antioxidants may also be considered as reducing RSS, although one has to be extraordinarily careful here. Many of these antioxidants, such as diallyltrisulfide (DATS) or diallyltetrasulfide (DATTS), do not act as reducing agents at all, but oxidize certain cysteine proteins, such as Keap-1, thereby triggering cellular signalling cascades which eventually lead to an—indirect—antioxidant response [39,40].

A somewhat special case is put forward by the class of persulfides (RSSH), which can be formed in proteins and, together with the trisulfanes (RSSSR’) may be more widespread in Biology than thought previously. The hunt for the reactive sulfane sulfur species is therefore truly heating up (see above), yet it is still not entirely clear if such RSSH species act primarily as oxidizing or reducing sulfur species, or, depending on the circumstances, perhaps even as both. This field of research is truly challenging, bearing in mind that first conceptual publications in this field, such as “Trafficking in persulfides: delivering sulfur in biosynthetic pathways” by Eugene G. Mueller have appeared in the literature ten years ago [98].

Whilst this is certainly a controversial topic in biology, from a chemical point of view, inorganic polysulfides most definitely are truly amazing redox active molecules, able to act as selective oxidants as well as reductants, as (multi-dentate) ligands to many metal ions and metalloproteins, complex structural features and, as John Toohey and Arthur Cooper have just reemphasized, as flexible and excellent thiol and disulfide modifying agents [48] (Figure 4). Indeed, their review published in 2014 and entitled “Thiosulfoxide (sulfane sulfur): New chemistry and new regulatory roles in biology” already strongly suggests a revolution to come [48]. Notably, and here we have to pay tribute to advanced inorganic chemistry, those inorganic polysulfides are able to rearrange to rather unusual but highly reactive forms, such as the previously mentioned thiosulfoxides, and their impact on thiol and disulfide proteins will surely result in new regulatory roles in biology. Note that a somewhat similar thiol-thione tautomerism also exists in organic chemistry, for instance in the natural product ergothioneine, which can be transported into certain cells via its own transporter, yet such molecules are seemingly rare in biology [48,99,100].

### 5.5. RSS: Radical Sulfur Species as Pinnacle of Sulfur Redox Chemistry

The third line of investigation which may usher in a significant change of concepts in the field of inorganic chemistry is the issue of lost electrons. It has long been known that the formation of S-nitrosothiols from RSH and ^•^NO is not possible unless one electron is consumed by a third party. This has led to extensive speculation, ranging from three-molecular reactions (which are rare) taking place in certain compartments or molecular cages, to the idea that one of the reaction partners is modified before, for instance as RSOH or indeed as thiyl radical RS^•^ for sulfur or, alternatively, as NO^+^ or N_2_O_3_ for nitrogen. Similarly, the release of ^•^NO from RSNO apparently lacks or exceeds one electron and hence nucleophilic attacks and the involvement of species such as the nitroxyl anion (NO^−^) have been considered. This specific niche of sulfur-nitrogen redox biology has also been very fruitful from the perspective of inorganic RSS. Molecules such as HSNO and its various (short-lived) rearrangement products have been postulated and can be added to the growing list of inorganic RSS. Furthermore, this field of research has also raised once more the question recently posed by Christine Winterbourn, namely “Are free radicals involved in thiol-based redox signaling?” [27]. Indeed, it seems that the one-electron oxidation of thiols to thiyl radicals should be more straightforward compared to the traditional view involving a two-electron oxidation to, for instance, sulfenic acid. Furthermore, the thiyl radical is more reactive, hence resolving some kinetic issues. Ultimately, it also leads directly to products such as disulfides, sulfenic acids and RSNO without the need for a third reaction partner or any unknown electron transfer processes.

In the end, the different lines of investigation mentioned above are all still speculative, and the key publications representing them often end with a question mark. Nonetheless, there is undoubtedly something unknown regarding the importance of radical reactions, and even if it does not lead to a revolution, it will surely characterise new RSS, significantly enriching the field of inorganic RSS. Eventually, these studies have saved the inorganic side of RSS from oblivion. We are now able to name quite a few inorganic RSS on par with their ROS or RNS counterparts, and this is a major improvement to the situation 15 years ago, even if it complicates a comprehensive definition of all RSS.

## 6. Food for Thought

We will now depart from the traditional view on organic or inorganic species and turn towards a couple of more complex, and still controversial issues which may pave the way for some future RSS research. When pondering about the concept of RSS in more detail, we must also consider, as Toohey and Cooper call it, “regulatory roles in biology” [48].

So let us briefly contemplate the kind of sulfur compounds which really matter in (human) biology. The most obvious choice of sulfur species involves the ones formed inside the human body, either by the human cells or the numerous and diverse bacteria sheltered within the gastrointestinal tract. This may be followed by exogenous species, and here one may first consider food, perhaps also inhalation of SO_2_ and H_2_S and, eventually, xenobiotics such as sulfur-based medications.

### 6.1. RSS Formed Inside Human Cells

As far as regulatory roles are concerned, RSS formed in the body seem to be most interesting, and indeed most ROS and RNS discussed today in the context of cell signalling are endogenous reactive species formed by certain enzymes. Whilst plants and lower organisms produce a wide spectrum of RSS—mostly secondary metabolites designed to put off or even harm competitors or predators—animal and human cells do not seem to produce such a variety of RSS. Admittedly, GSSG and some of its more exotic derivatives, such as GSSG^•−^ or GS(O)SG are produced inside eukaryotic cells and probably on purpose, the same applies to lipoic acid, and it now seems that besides H_2_S, certain inorganic polysulfides and eventually also organic per- and polysulfanes may be formed endogenously. It is also possible that H_2_S meets ^•^NO (either directly or in the context of S-nitrosothiols) and that species such HSNO, as well as other products of H_2_S reactions with oxygen, ROS, RNS and RSS, may be formed inside the human body on a more regular basis.

Still, most of this kind of chemistry is exotic, rare and certainly not on par with ROS that can be found inside the human body. Perhaps the evolutionary circumstance that humans cannot reduce and oxidize easily along the sulfur (or indeed nitrogen) pathways may serve as an explanation, and one may also point the finger into the direction of our aerobic lifestyle, which some believe has compromised our ability to play as virtuosically with sulfur as we play with oxygen.

The issue becomes somewhat foggy when we turn our attention towards the numerous reactive sulfur modifications in proteins in human cells. Apart from lipid peroxides, the concept of ROS normally does not encompass such large-molecule based reactive species. Hence should we consider all those amazing sulfur modifications in proteins, which have been identified by extensive, often cumbersome studies over the last few years, as RSS? A look at a possibly similar kind of protein chemistry in the case of oxygen or nitrogen, of ROS and RNS, respectively, remains surprisingly fruitless. Neither alcohols nor amines are usually involved in redox signalling, as they are not the prime targets attacked by the milder forms of oxidative stressors (although aldehyde formation is possible). Aromatic amino acids can be modified, for example nitrotyrosine or dityrosine formation by RNS. Yet these oxidized amino acids are not thought to be involved in signalling and, in any case, there is no switching step, as such oxidative modifications, if and when they occur, are generally irreversible. Hence sulfur is a special case. Bearing in mind that such protein modifications are secondary, i.e., reaction products formed by a preceding onslaught of ROS and RNS on cysteine residues which are the initial primary targets of reactive species, we would rather consider them in the context of switching and signalling—and probably not as RSS in their own right.

### 6.2. The Culinary Side of RSS: Organic Sulfur Compounds

We would therefore argue that most of the reactive sulfur species which deserve the RSS predicate are small molecules. In the context of the human body, such RSS appear to be primarily derived from endogenously synthesized sulfides, either H_2_S or molecules such as GSH and lipoic acid, and often by oxidation. In contrast, exogenous RSS form a rich and diverse field of secondary metabolites in plants and lower organisms. In this regard, RSS once more differ from ROS (and RNS) as they have the potential to transduce species barriers, conveying chemistry found in the bacterial, fungal and plant kingdoms into the mammalian realm.

**Figure 5:**
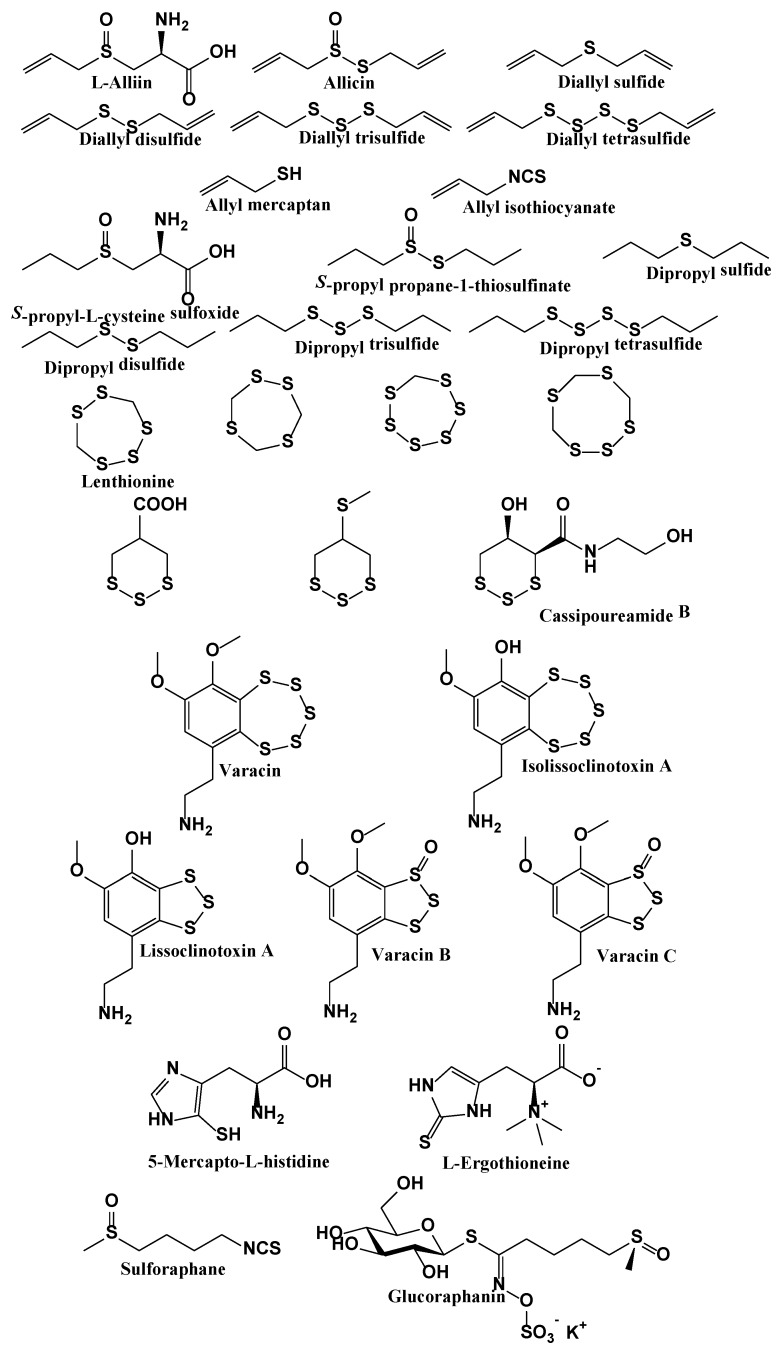
Overview of selected sulfur containing natural products found in edible plants, mushrooms and some lower organisms. These reactive sulfur secondary metabolites have been associated with all kinds of health benefits and play a major role in so-called functional foods, especially for the elderly. In most cases, these claims are controversial because of concentrations, bioavailability and stability. Still, a specific interaction with the enzymes and microbiota of the gut and subsequent formation of “reprocessed sulfur species” may be relevant. If and how these substances act as RSS in a more narrow biological sense is a matter of ongoing research. See text for details on the origin and potential uses of these suspected nutraceuticals.

Even carnivores on occasion eat plants, and hence come in contact with those secondary metabolites. Apart from environmental toxins such as SO_2_, we therefore find a particularly rich menu of OSCs in our daily nutrition, spiced up by some of the sulfur metabolites formed in the gut. Indeed, humans consume considerable amounts of sulfur each day, and some of this sulfur is in the form of RSS. Figure 5 provides a brief overview of the more culinary side of RSS, and highlights some of the regulatory roles in biology associated with them. It should be noted that a fair number of these RSS are not stored in the organism they originate from, yet are formed in situ from a precursor and an enzyme designed to arm them as part of an intricate defence system [101]. Such bi-component (cyto-)toxic systems able to generate RSS on demand are currently inspiring research in Pharmacy and Agriculture [102,103].

There is, of course, the old argument that many of those species may not really matter in the context of human biology as they cannot really enter the body, let alone individual cells. This argument of bioavailability needs to be taken seriously, yet some of these compounds, such as allicin, seem to diffuse readily across membranes, whilst others, such as ergothioneine, actually feature their own transporter in some human cells, which turns them into relevant, readily available and, above all, also selective endogenous RSS to consider for human consumption [100,104]. In any case, the sulfur we consume orally at some stage ends up in the gastrointestinal tract, and even if some species cannot cross into the blood stream, they can still direct an impressive and diverse orchestra of gut bacteria.

### 6.3. Reprocessed Sulfur Species Produced by the Microbiota

Turning towards digestion, it is likely that the food-based RSS such as allicin, mustard oil, diallylsulfides and isothiocyanates represent just the tip of the iceberg of the kind of RSS which are subsequently being formed from them in the gut by intestinal bacteria. It is a fact that the gut microbiota extensively metabolize the diverse sulfur compounds we consume throughout the day and eventually reach the intestine. Those bacteria differ in their ability to oxidize, and especially also to reduce, sulfur compounds. Eventually, some of this sulfur may end up in form of H_2_S, as has been demonstrated already in various scientific studies [105]. For instance, Ruma Banerjee and her colleagues have recently discussed “Sulfur as a signaling nutrient through hydrogen sulfide”, and in their publication clearly emphasize the importance of H_2_S as the central species of (nutritional) sulfur metabolism [106]. The issue of RSS in food has also indirectly given rise to some rather curious names for popular culinary dishes, such as the traditional German “hand cheese with music”. Yet H_2_S is probably only one of many RSS formed by the microbiota. Considering the multitude of different bacteria inhabiting the gut at a time, it is likely that they indeed generate and release a wide range of RSS, most of whom have probably so far escaped detection or our attention. Hence the idea of sulfur as a signaling nutrient with important physiological and possibly also therapeutic attributes holds considerable attraction.

Within this context, one must also bear in mind that such sulfur species have the potential to alter the composition of the microbiome. Many RSS, such as allicin and diverse polysulfanes, exhibit pronounced antimicrobial activity, and it is probable that their presence or formation in the gut will impact considerably on the medley of the bacteria found there [95,107,108,109]. Eventually, a certain cycle of RSS controlling the composition of gut bacteria, which in turn determine the kind of RSS formed, may emerge, yet those matters are complicated, most likely depend on the host organism, its age and—nutritional—habits and in any case need to be investigated more thoroughly in the future.

Here, one must also remember that not all sulfur species are healthy and that there is no argument for an excessive consumption of sulfur. A recent publication by Peter Stroot in “Medical Hypotheses” highlights this often forgotten aspect of RSS research [110]. Whilst surely controversial, the author claims that “The primary cause of oxidative stress is ultra-exogenous sulfide formation (USF)” [110]. Yet this matter is indeed more serious, as considerable amounts of sulfate (SO_4_^2−^), which itself is clearly not an RSS, and some sulfite (SO_3_^2−^) are consumed each day. It would be most intriguing to investigate which RSS are formed from such sulfur compounds by bacteria directly, and indirectly after H_2_S is produced and subsequently becomes oxidized again to reactive sulfane sulfur species, ranging from oxidized polysulfide products to the protein modifications (persulfides, polysulfanes) which are associated with them. The interplay of different gut bacteria, in concert with human cells, is likely to give rise to a wide variety of RSS, most of them probably inorganic species or modifications of small thiol-containing molecules, GSH, proteins or enzymes.

### 6.4. RSS: Recreational Sulfur Species

**Figure 6:**
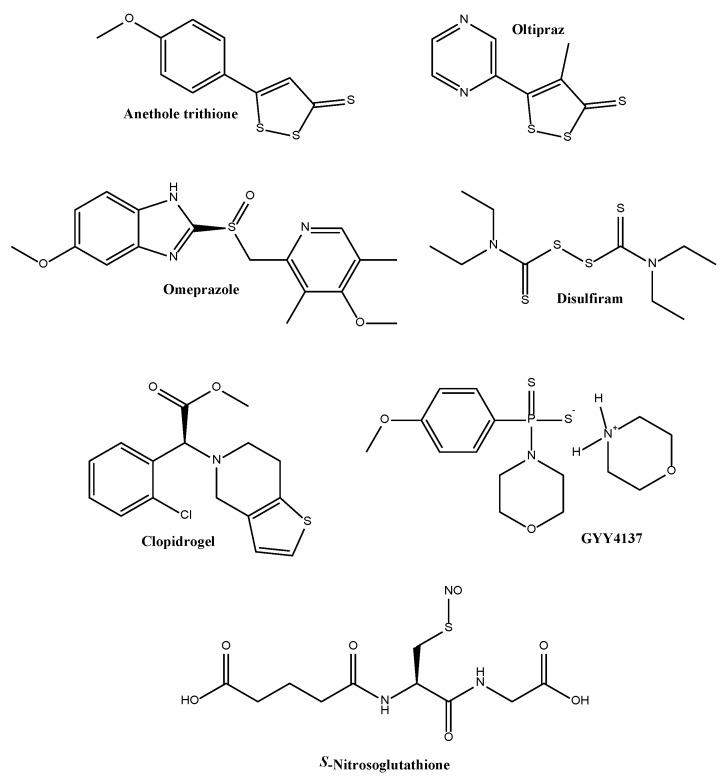
Several organic, sulfur-containing compounds are used as food supplements or medical drugs. In some instances, such as disulfiram, the nature of a RSS is immediately apparent. Other compounds, such as omeprazole or clopidogrel, become activated and react via a reactive sulfur intermediate, yet their classification as RSS drug is less apparent. The same applies to releasing agents, for instance GYY4137, which harbour certain RSS, such as H_2_S. Still others, such as the 1,2-dithiole-3-thiones, are under investigation or controversial with regard to their medical or recreational uses.

The gut may therefore provide an interesting back door for the formation and activity of an amazing variety of reactive RSS inside humans, but not by human cells. Still, we should briefly return to the upper intestinal tract. Here, a stringent smell of sulfur from the mouth is usually not the result of metabolism by bacteria in the oral cavity, but by exhalation of sulfur compounds via the lung. In the other direction, we often find a flow of odd sulfur-based remedies, such as Haarlem Oil, which are sold freely as traditional medicines and are, among others, used to dope horses and, in all seriousness, intellectuals [111]. Many of the more or less active ingredients in these supplements may be considered as RSS. Figure 6 provides an overview of such RSS-containing substances which are used either to promote health or indeed as accepted drugs.

In fact, there is a rather profound piece of underlying chemistry associated with many of these remedies. Most of them act via the modification of critical thiol residues in their respective target proteins, and this may result in widespread cellular signalling and feedback, as already mentioned in the context of Keap-1 modification, nuclear factor 2 (Nrf2) release and a subsequent antioxidant defence. Certain medical drugs, such as omeprazole and structurally related prazoles, also seem to function via such an oxidative mechanism [112,113]. Omeprazole is a proton pump inhibitor targeting a potassium dependent ATPase. The sulfoxide is used in the therapy of various gastrointestinal ulcers and becomes activated under acidic conditions to form a reactive sulfenic acid. The latter then reacts with a cysteine residue in the ATPase to form a disulfide, a process which irreversibly shuts down the enzyme and causes an increase in stomach pH. Other agents, such as clopidogrel, a drug used to prevent thrombosis, also become activated under certain conditions to form a reactive RSS which subsequently modifies specific target proteins via a disulfide bond. In the case of the antiplatelet agent clopidogrel, the reactive intermediate is not well defined in most literature on this topic, yet regardless of its exact nature it is able to modify P2Y12 and hence inhibit blot clotting and thrombosis. In contrast, 1,2-dithiole-3-thiones, such as the schistosomicide oltipraz, react without any prior activation. This compound, together with a range of structurally related 1,2-dithiole-3-thiones, can react via a cascade of—mostly oxidizing—chemical attacks on thiol targets and has also been linked to the formation of oxidative stress [114]. The link between RSS and increased levels of oxidative stress is far from surprising, as RSS often consume reduced thiols and hence directly or indirectly lead to an increase of ROS concentrations. This kind of selective oxidation can be exploited in the therapy against microbes and is also under consideration in the context of cancer therapy [39,40,107,108,115]. Recent studies involving a wide spectrum of 1,2-dithiole-3-thiones have associated some of these sulfur compounds with the induction of cytoprotective Phase 2 enzymes, but also with the inhibition of histone deacetylase enzymes [116,117]. Other RSS, such as DATTS from garlic have delivered stunning results in the context of systemic sclerosis (or scleroderma, SSC), and as described above, may even be used to immunize against oxidative stress by triggering antioxidant defence pathways, an action particularly useful in the elderly [118].

Indeed, many natural, mostly plant based RSS are found in traditional medicine, together with sulfur-based antioxidants. Remedies such as Haarlem Oil function primarily via RSS, and this comes as no surprise as this particular mixture harbours considerable amounts of 1,2-dithiole-3-thiones. Besides these more exotic examples, there has been a recent surge in interest in H_2_S releasing drugs, for instance by Peter Moore and colleagues [119]. Despite the fact that there may still be some issues related to mistaken identities, research into the “Synthesis and biological effects of hydrogen sulfide (H_2_S): development of H_2_S-releasing drugs as pharmaceuticals” is likely to grow and to prosper during the coming years [106,120]. Eventually, there are a few recent reviews which touch upon this matter and may be consulted for additional information and further inspiration.

## 7. Towards a Modern Definition of RSS

After this rather demanding tour de RSS, we will now return to the initial signpost and the question of the genuine nature of RSS in Biology. 15 years ago, matters were far more simple, with less species and interest around, and our initial definition of RSS was seen in the context of “the potential of sulfur in higher oxidation states to be formed under conditions of oxidative stress, and for these reactive sulfur species (RSS) to act as further aggressive oxidizing agents” [1].

It is quite surprising that this definition has not changed much during the last 15 years, despite the fact that many new and unorthodox molecules have entered the scene and there has been a true revolution in our understanding of sulfur redox biology. A more recent definition of RSS by Robert Brannan in 2010 is more or less identical to the original one: “Reactive sulfur species (RSS) are redox-active sulfur compounds formed under conditions of oxidative stress that may be capable of initiating oxidation reactions” [121].

Yet glancing over the publications we have just reviewed, the initial definition of RSS appears to be very restricted and, as we admit freely, is probably no longer appropriate. 15 years ago, the definition of RSS focussed on the rather narrow biological condition of oxidative stress and here primarily on oxidizing RSS generated as secondary oxidative stressors under oxidative stress, and as part of more extensive oxidation cascades. Countless RSS, from secondary metabolites in plants and present in our daily food, to metabolized sulfur species, H_2_S and its derivatives and all the way to drug-like RSS in ointments and used for therapy are clearly not covered by this narrow definition. Table 2 summarizes and comments some of these unorthodox interpretations of the RSS concept.

It is embarrassing, yet we have to admit that the definition of RSS on Wikipedia is probably more revealing and appropriate, despite the fact that RSS cannot “be” a family, but may “form” one.

“Reactive sulfur species (RSS) are a family of sulfur-based chemical compounds that can oxidize and inhibit thiol-proteins and enzymes. They are often formed by oxidation of thiols and disulfides into higher oxidation states. Examples of RSS include persulfides, polysulfides and thiosulfate” [122].

Whilst this definition no longer exclusively focuses on RSS as products of oxidative stress, and also mentions some of the prime targets of RSS in proteins and enzymes, it still seems to exclude a variety of important players, from the organic RSS and reducing RSS all the way to species that interact with targets other than protein-bound cysteine thiols. In a 2012 publication Gruhlke and Slusarenko discussed that several RSS are not produced as a result of oxidative stress per se and that some are oxidizing (e.g., RSOH) and some are reducing (e.g., RSH, H_2_S). This publication also expanded the variety of targets and functions or consequences, and offered the following definition of RSS as “redox-active sulfur-containing molecules that are able, under physiological conditions, to either oxidize or reduce biomolecules” [5].

This definition is not completely satisfying either and does not easily lead to a succinct list of RSS species, which would be very desirable. Still, a reworking for more precision could improve this definition. For example, concentrating more on functional groups, rather than just on sulfur-containing molecules, would be an improvement. Thus, each individual thiol (RSH) and each individual disulfide (RSSR’) would, according to the above definition, be categorized as an RSS in its own right. Categorizing on the basis of the sulfur-containing redox-active functional groups rather than sulfur in general negates the problem. In that case, thiols and disulfides would represent specific RSS categories. Still, since the majority of current interest in this area seems to be in the biological and medical fields, it is felt important to retain the “under physiological conditions” part of the definition. At the same time, it also seems to be important to emphasize the wider impact of RSS under those conditions, as we wish to rule out species or amounts of species that go unnoticed.

Eventually, we therefore arrive at a more timely and practical hypothesis for RSS which we will propose as working definition for future discussion: “RSS are defined as those molecules which contain at least one redox-active sulfur atom or sulfur-containing functional group in their structure and are capable of either oxidizing or reducing biomolecules under physiological conditions to trigger or propagate a noticeable cellular signal or wider biological event.”

## 8. Conclusions and Outlook

The previous sections have considered many aspects of the rather extensive family of RSS, which has grown during the last 15 years from a handful of exotic species to a roll-call of numerous different sulfur modifications, many of which are now firmly anchored within the mainstream of redox biology. These RSS feature highly in the context of the emerging “redox code” as proposed recently by Dean Jones and Helmut Sies [123]. Travelling back to 2001, one would hardly have expected at the time that certain lesser known modifications, such as the sulfenic acids or inorganic polysulfides, would soon be at the centre of extensive mining activities and pursuits for mistaken identities, eventually giving rise to entire signalling cascades, sulfenomes and scientific revolutions. Similarly, who would have expected then that some exotic OSCs from more or less exotic plants would turn out to be attractive biologically active nutraceuticals, that sulfur would end up as a signalling nutrient at the centre of healthy eating, for instance in the elderly or intellectuals, and that certain RSS may control the microbiome (and vice versa)? Today, the field of sulfur redox biology is firmly established, with all its different variations ranging from human redox biology to plant metabolites, medical and agricultural uses, and with all its exciting small but vital niches.

It is now time to turn our attention to the next 15 years, which no doubt will bring with them considerable excitement and changes. In order to conclude, we will try to catch some of the wind of change and try to identify the next generation of sulfur redox chameleons coming our way.

### 8.1. Diversification and Fragmentation

First of all, the next 15 years will probably witness a more diversified scientific scene dealing with rather different RSS and concepts thereof. We therefore anticipate not only a further rise of RSS and RSS research, but also a continued diversification and ultimately also fragmentation of the field. Generally, such developments simply reflect the coming of age of a particular area of science, and therefore are of little concern. No one should be astonished, for instance, if the field of mammalian sulfur redox biology would eventually forge closer ties with organic chemists, medicine and pharmacy, and leave the yeasts behind.

Similarly, the theme of RSS and nutrition, of research into health foodstuffs for the elderly, will probably grow considerably as Western Societies are ageing and nutrition becomes ever more important. Here, a particular interest will also reside on how to control the microbiota with *Allium* species, such as garlic and onions, and other culinary products rich in OSCs. Plant biologists will, of course, cherish these developments and intensify their search for RSS or RSS releasing agents as secondary metabolites in plants, fungi and other microbes. Likewise, from a pharmaceutical perspective, there is increasing interest in developing drugs which generate RSS (or modulate current RSS signalling) with the promise of developing new drugs for the treatment of conditions such as inflammation, cardiovascular disease and cancer [124,125].

### 8.2. New Players on the Block

Whilst the different communities involved in RSS will grow and diversify, we are also likely to experience a more diversified range of molecules sheltering under the umbrella of RSS, with their own chemistry and biological activity. It is likely, for instance, that the hunt and eventually mining for RSS, reactive species and intermediates in the cell and post-translational modifications in proteins will intensify. The current debate on the genuine identity of the active species involved in the biological action of H_2_S in humans provides a foretaste of such developments. It is almost certain that the field will not pause with HSNO, SSNO^−^, or S_x_^2−^. Other species, such as partially oxidized forms of H_2_S or H_2_S_x_, for instance HSOH, HOS_x_OH, H_2_S_x_O_y_, and certain peroxo-species, such as RSOOH will emerge or experience a certain renaissance, maybe in form of Reactive Sulfur Oxygen Species (RSOS) or as Reactive Sulfur Nitrogen Species (RSNS), or both (RNSOS), in analogy to the Reactive Nitrogen Oxygen Species (RNOS) which populate the field already. In their recent publication, Toohey and Cooper have already identified a few interesting inorganic species, and more are likely to emerge [49]. Here, thiosulfoxides are a harbinger of such chemically and biochemically challenging developments to come.

At the same time, more unusual post-translational cysteine and methionine modifications are about to enter the scene. The recent claims that trisulfanes can be found in proteins more widely than previously thought likely foreshadow more surprises to come. We need to remember once more that such unusual modifications are hard to pin down inside cells, as they have no unique physical or chemical properties which may be used to detect and to distinguish them from other modifications. We can therefore expect quite a few controversies in that field and proteome mines to be dug and to collapse before the dust has settled. Yet, once again, the previous 15 years have also been controversial, and we would not be surprised if 15 years from now, colleagues were to refer routinely to the cell’s sulfoxidome or trisulfanome.

Talking about metamorphosis, the recent renaissance of selenium research may encourage us to somewhat plagiarize our initial concept of RSS and apply it in the context of Reactive Selenium Species (RSeS). Indeed, such a concept may be useful to cover: (a) newly identified natural selenium metabolites, such as selenoneine, a rather unusual natural selenium compound which has been found in the blood of tuna and seems to be highly reactive, (b) inorganic selenium species with biological relevance, such as naturally generated selenium nanoparticles found in many strains of *Staphylococcus* and other bacteria and used on occasion to fortify drinks with selenium, (c) selenium agents currently in development with potential pharmaceutical applications, such as organic seleninic acids, organic selenourea derivatives, selenazolinium salts, and (d) recently discovered selenoproteins and the various modifications of selenocysteine found already or to be expected in those proteins (such as selenosulfide, diselenide, seleninic acids, selenoxides, etc.) [126,127,128,129,130,131,132,133,134,135,136,137,138,139,140,141,142]. Whilst those RSeS at first sight may be less relevant to redox biology compared to RSS because of their number, variety and concentration in most cells and organisms, they are clearly on the rise in the field of food supplementation and pharmaceutical research. Not all of these RSeS stand in the tradition of ebselen by acting as antioxidants, and some exhibit rather more diverse, often oxidizing, and hence cytotoxic actions. These may come to have important pharmaceutical and agricultural applications, for instance as fungicides, nematicides or selective anticancer agents.

Eventually, the different strands of RSS and related chalcogen research will continue to grow and to prosper—and probably demand a further revision of the current concept during the years to come. Hence we will terminate our discussion here and we will be back—in 2031.

## Figures and Tables

**Table 1:** Reactive Sulfur Species as defined in 2001.

Reactive Species	Chemical Formula	Oxidation State(s) of Sulfur
Thiyl radical	RS^•^	−1
Disulfide	RSSR	−1
Disulfide-*S*-monoxide (thiosulfinate)	RS(O)SR’	+1, −1
Disulfide-*S*-dioxide (thiosulfonate)	RS(O)_2_SR’	+3, −1
Sulfenic acid	RSOH	0
Sulfinic acid	RSO_2_H	+2

This table has been adapted from “Table 2” of our original article [1] and also includes some entries which at the time where mentioned in the text, but not shown in the original Table. The formal oxidation state of R is assumed to be +1.

**Table 2:** Summary of various uses of the RSS concept, often by different communities. Please note that some of these names have indeed been used in the literature as the genuine unabbreviated form of RSS, whilst others have been devised by us to emphasize particular uses or clusters of compounds.

Interpretation of RSS	Examples
Reactive sulfane species	RS_x_R’, RS_x_H (R ≠ H, x ≥ 2)
Reducing sulfur species	H_2_S, GSH, protein bound thiols, under specific circumstances possibly S_x_^2−^
Radical sulfur species	Primarily RS^•^, GSSG^•−^, R’SR^•+^, HS^•^
Reactive sulfide species	H_2_S_x_ and its deprotonated forms
Reprocessed sulfur species	Biologically active sulfur metabolites formed by microbiota
Reactive sulfur substances	SO_2_, SO_3_, S_2_O_3_^2−^, H_2_SO_5_ (often with limited biological relevance)
Reactive sulfur secondary metabolites	Allicin, DADS, DATS, DATTS
Recreational sulfur substances	Haarlem oil, 1,2-dithiol-3-thione
Regulatory sulfur species	Disulfides, sulfenic and sulfinic acids in proteins and enzymes as part of signalling and control

## Abbreviations

DADS; Diallyl disulfide, DATS; Diallayl trisulfane, DATTS; Diallyl tetrasulfane, GFPs; Green Fluorescent Proteins, GSH; Glutathione, Nrf2; Nuclear factor 2, OSCs; Organic Sulfur Compounds, Prdx; Peroxiredoxin, RSS; Reactive Sulfur Species, RSeS; Reactive Selenium Species, ROS; Reactive Oxygen Species, RNS; Reactive Nitrogen Species, SSC ;systemic sclerosis, Srx; sulfiredoxin , YFPs; yellow fluorescent proteins.
